# Apamin Suppresses LPS-Induced Neuroinflammatory Responses by Regulating SK Channels and TLR4-Mediated Signaling Pathways

**DOI:** 10.3390/ijms21124319

**Published:** 2020-06-17

**Authors:** Jihyun Park, Kyung Mi Jang, Kwan-Kyu Park

**Affiliations:** 1 Department of Pathology, College of Medicine, Catholic University of Daegu, Daegu 42472, Korea; jihyunp@cu.ac.kr; 2 Department of Pediatrics, Yeungnam University College of Medicine, Daegu 42415, Korea

**Keywords:** neuroinflammation, CaMKII, TLR4, apamin, NF-κB, STAT3, MAPK-ERK

## Abstract

Neuroinflammation plays a vital role in neurodegenerative conditions. Microglia are a key component of the neuroinflammatory response. There is a growing interest in developing drugs to target microglia and thereby control neuroinflammatory processes. Apamin (APM) is a specifically selective antagonist of small conductance calcium-activated potassium (SK) channels. However, its effect on neuroinflammation is largely unknown. We examine the effects of APM on lipopolysaccharide (LPS)-stimulated BV2 and rat primary microglial cells. Regarding the molecular mechanism by which APM significantly inhibits proinflammatory cytokine production and microglial cell activation, we found that APM does so by reducing the expression of phosphorylated CaMKII and toll-like receptor (TLR4). In particular, APM potently suppressed the translocation of nuclear factor kappa-light-chain-enhancer of activated B cells (NF-κB)/signal transducer and activator of transcription (STAT)3 and phosphorylated mitogen-activated protein kinases (MAPK)-extracellular signal-regulated kinase (ERK). In addition, the correlation of NF-κB/STAT3 and MAPK-ERK in the neuroinflammatory response was verified through inhibitors. The literature and our findings suggest that APM is a promising candidate for an anti-neuroinflammatory agent and can potentially be used for the prevention and treatment of various neurological disorders.

## 1. Introduction

Neuroinflammation plays a vital role in the etiology and progression of neurodegenerative diseases including ischemic stroke, Alzheimer’s disease (AD), and Parkinson’s disease (PD) [1]. Microglia, the resident immune cells of the central nervous system (CNS), play an important role in neuroprotection and are involved in various neurodegenerative pathologies [2]. Microglia respond to CNS damage by up-regulating functions that involve Ca^2+^ signaling; proliferation; migration; phagocytosis; and the production of nitric oxide, interleukins, cytokines, and chemokines [3]. Activated microglia produce neuroinflammatory responses by releasing various proinflammatory cytokines and mediators including tumor necrosis factor α (TNFα), interleukin 1β (IL1β), interleukin 6 (IL6), and cyclooxygenases-2 (COX2) [4]. These neuroinflammatory responses are strongly correlated with neurodegenerative diseases and lead to synaptic degeneration, neuronal cell death, and cognitive dysfunction [5]. Lipopolysaccharides (LPS) increase the proinflammatory cytokines of microglia by up-regulating the small conductance calcium-activated potassium (SK) channel, toll-like receptor (TLR4) expression, phosphorylated mitogen-activated protein kinases (MAPK) phosphorylation, and transcription factor translocation [6,7,8]. The increased neuroinflammatory response activates the ability to kill neurons through a peroxy-nitrite-mediated mechanism [7]. Accordingly, the modulation of neuroinflammatory responses represents a potential therapeutic strategy for a wide range of pathological conditions, including neurodegenerative diseases.

The role of microglia-mediated neuroinflammation in the progressive nature of many neurodegenerative disorders has been demonstrated in models of microglial cells activated by proinflammatory stimuli [5]. Microglia are sentinels for LPS-mediated inflammation and are involved in both innate and adaptive immune responses in the CNS [9]. The interaction between LPS and TLR4 activates inflammation-associated transcription factors, such as nuclear factor kappa-light-chain-enhancer of activated B cells (NF-κB) and signal transducer and activator of transcription (STAT) [10,11,12,13,14,15]. In addition, studies have revealed that the transcription and production of proinflammatory factors in activated microglial cells involve MAPKs [14,16,17,18]. Recently, we reported potential candidates for the treatment of neurodegenerative disorders through the inhibition of neuroinflammation and neurotoxicity blocking of NF-kB-STAT3 and MAPK-extracellular signal-regulated kinase (ERK) in neuroblastoma cell lines and primary mesencephalic neurons [19]. Thus, inhibiting NF-κB, STAT, and MAPKs pathways may be promising approaches to combat the progression of neurodegenerative processes associated with inflammation and microglial activation.

Apamin (APM), a specifically selective antagonist of the SK channels, is an 18-amino-acid peptide found in apitoxin [20,21]. Our previous studies have confirmed that APM is an anti-inflammatory and antiapoptotic agent that acts through vascular smooth muscle cell migration [22], biliary fibrosis and activation of hepatic stellate cells [23], TNFα/interferon (IFN)γ-induced keratinocytes [24], LPS/fat-induced atherosclerotic mice [25], and macrophage activation [26]. In addition, we recently investigated and reported the effect of APM on nonneoplastic disease [27]. Although studies have investigated the physiological function of APM, including anti-inflammatory and antiapoptotic functions, the effectiveness of APM related to LPS-induced microglial neuroinflammation has not been evaluated.

Therefore, in this study, we investigated the potential therapeutic effects of APM on the production of proinflammatory cytokines induced by LPS. We also evaluated the molecular impact of APM on the signal transduction pathways involved in LPS-induced neuroinflammatory responses in BV2 microglial cells and primary microglial cells.

## 2. Results

### 2.1. APM Significantly Decreased LPS-Induced Proinflammatory Cytokines in Microglial Cells

LPS-induced proinflammatory cytokine production was performed using cultured microglial cells according to a previously described method [28]. To investigate the influence of TNFα on LPS-induced microglial activation, BV2 cells were treated with different concentrations of LPS for 12 h and analyzed by an established Enzyme-Linked Immunosorbent Assay (ELISA) assay. The secretion of TNFα significantly increased LPS-stimulated BV2 microglial cells in a concentration-dependent manner (Figure S1A). This increase was confirmed through an immunoblotting assay (Figure S1B). We also determined the expression levels of CD11b in LPS-stimulated BV2 microglial cells. LPS significantly stimulated CD11b expression in a concentration-dependent manner. When exposed to 1 μg/mL of LPS, BV2 cell activation and proinflammatory cytokine secretion significantly increased.

Before investigating the pharmacological potentiality of APM, its cytotoxic effects on BV microglial cells were examined through a CCK assay. The treatment of cells with 0.5–5 μg/mL of APM was not cytotoxic and mildly inhibited growth, with a 10% decrease in cell proliferation at 1 μg/mL (Figure S1C). Thus, APM was used at a concentration of 1 μg/mL for subsequent experiments.

**Figure 1:**
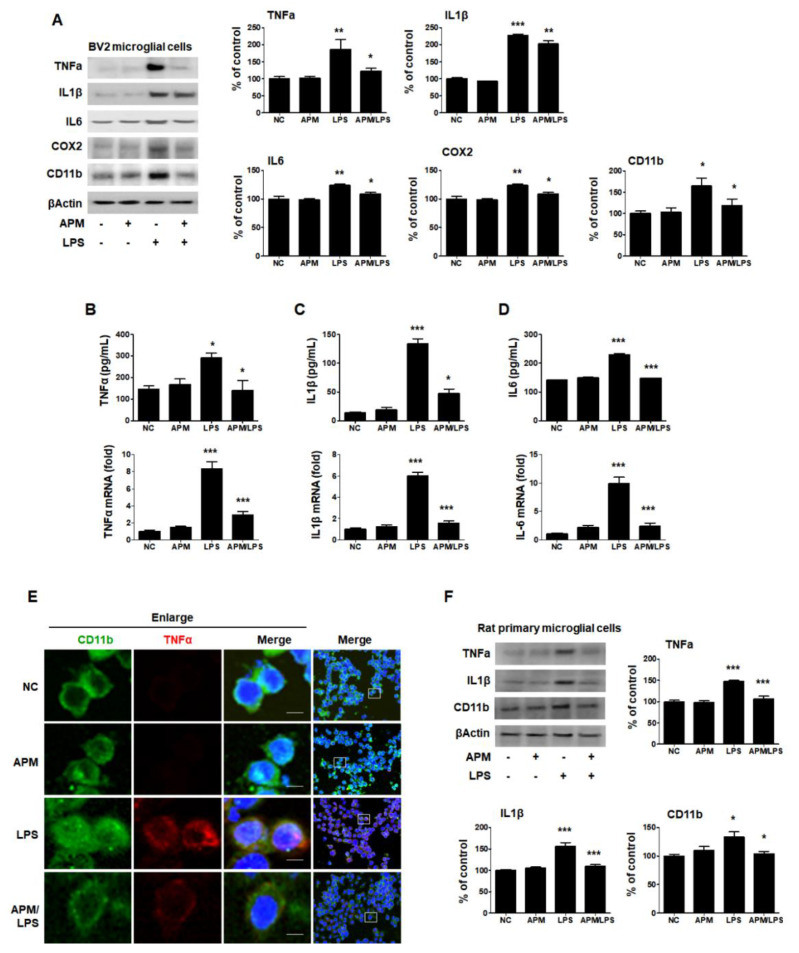
APM inhibits LPS-induced proinflammatory responses in LPS-stimulated BV2 and rat primary microglial cells. Cells were incubated in the presence or absence of APM for 1 h and then treated with LPS for 12 h. CD11b, TNFα, IL1β, IL6, and COX2 expression was significantly inhibited in LPS-stimulated BV2 microglial cells by APM administration (**A**). APM strong suppressed LPS-induced TNFα (**B**), IL1β (**C**) and IL6 (**D**) production both in extracellular and mRNA levels. Immunofluorescence double staining for CD11b (green) and TNFα (red) localization in BV2 microglial cells (**E**). Cell were counterstained with DAPI (blue). Magnification 400×. Enlarge figure of scale bars: 5 μm. APM strong suppressed LPS-induced TNFα, IL1β and CD11b expression in rat primary microglial cells (**F**). βActin was used to confirm equal sample loading. TNFα, IL1β, IL6, COX2, and CD11b followed by densitometric analysis. The data are representative of three independent experiments and quantified as mean values ± SEM. Tukey’s multiple comparison test, * *p* < 0.05, ** *p* < 0.01, *** *p* < 0.001 compared to normal control.

Therefore, the inhibitory effects of APM on microglial activation and the proinflammatory cytokine response induced by LPS and BV2 cells were treated with APM for 1 h, followed by 1 μg/mL of LPS for 12 h. APM alone did not alter any proinflammatory cytokine expression compared with vehicle treatment without LPS (Figure 1A). APM significantly suppressed the LPS-induced CD11b, TNFα, IL1β, IL6, and COX2 expression. Next, we examined the LPS-stimulated release and mRNA level of TNFα, IL1β, and IL6 after APM treatment and found that APM strongly suppressed their release and inhibited their mRNA expression (Figure 1B–D).

To further confirm our findings, we observed the subcellular localization of CD11b and TNFα. Consistent with the protein, mRNA level, and ELISA results, APM significantly down-regulated LPS-induced TNFα expression in BV2 cells (Figure 1E). Lastly, we examined whether APM alters LPS-induced proinflammatory responses in rat primary microglial cells. Rat primary microglial cells were treated with APM for 1 h followed by LPS for 12 h, and immunoblotting was performed (Figure 1F). Increased TNFα, IL1β, and CD11b expression were significantly inhibited in LPS-stimulated rat primary microglial cells by APM treatment. Thus, these data suggest that APM treatment regulates the activation of microglial cells by LPS stimulation and their proinflammatory production.

### 2.2. APM Strongly Inhibited LPS-Induced SK2 Channels in BV2 Microglial Cells

**Figure 2:**
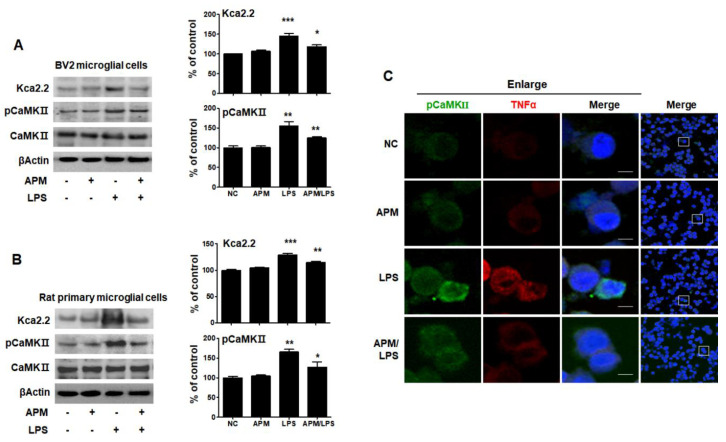
APM inhibits LPS-induced SK channels in BV2 and rat primary microglial cells. Cells were treated with APM for 1 h followed by LPS for 6 h. APM significantly inhibit LPS-induced K_Ca_2.2 and pCaMKⅡ expression in BV2 (**A**) and rat primary microglial cells (**B**). Immunofluorescence double staining for pCaMKⅡ (green) and TNFα (red) localization (**C**) in BV2 microglial cells. Cell were counterstained with DAPI (blue). Magnification 400×. Enlarge figure of scale bars: 5 μm. βActin was used to confirm equal sample loading. K_Ca_2.2 and pCaMKII followed by densitometric analysis. The data are representative of three independent experiments and quantified as mean values ± SEM. Tukey’s multiple comparison test, * *p* < 0.05, ** *p* < 0.01, *** *p* < 0.001 compared to normal control.

APM has long been known as a specifically selective blocker of SK2 channels [27]. Ca^2+^/calmodulin-dependent protein kinase II (CaMKII), one of the main downstream targets of Ca^2+^ and CaM, is activated by Ca^2+^/CaM [29]. TNFα is produced in SK2/K_Ca_2.2 channel-activated microglia [8]. To examine whether APM itself can regulate the SK2/K_Ca_2.2 channel, BV2 and rat primary microglial cells were treated with APM for 1 h followed by LPS for 6 h, and immunoblotting was conducted with anti-K_Ca_2.2 and CaMKII antibody. The expression of LPS-induced K_Ca_2.2 and pCaMKII significantly increased compared with normal control, respectively (*p* < 0.001, *p* < 0.01). APM itself significantly inhibited LPS-induced K_Ca_2.2 (*p* < 0.05) and pCaMKII (*p* < 0.01) expression in BV2 microglial cells (Figure 2A). These results are consistent with LPS-induced rat primary microglial cells (Figure 2B). To further confirm our findings, we observed the subcellular localization of pCaMKII and TNFα expression (Figure 2C). As expected, APM significantly decreased LPS-induced subcellular localization of pCaMKII and TNFα expression in BV2 microglial cells. Our results suggest that APM itself directly inhibits LPS-induced SK2/K_Ca_2.2 expression. Thus, a decrease in the subcellular localization of pCaMKII and TNFα expression observed.

### 2.3. APM Regulates TLR4 to Alter LPS-Induced Proinflammatory Cytokines

**Figure 3:**
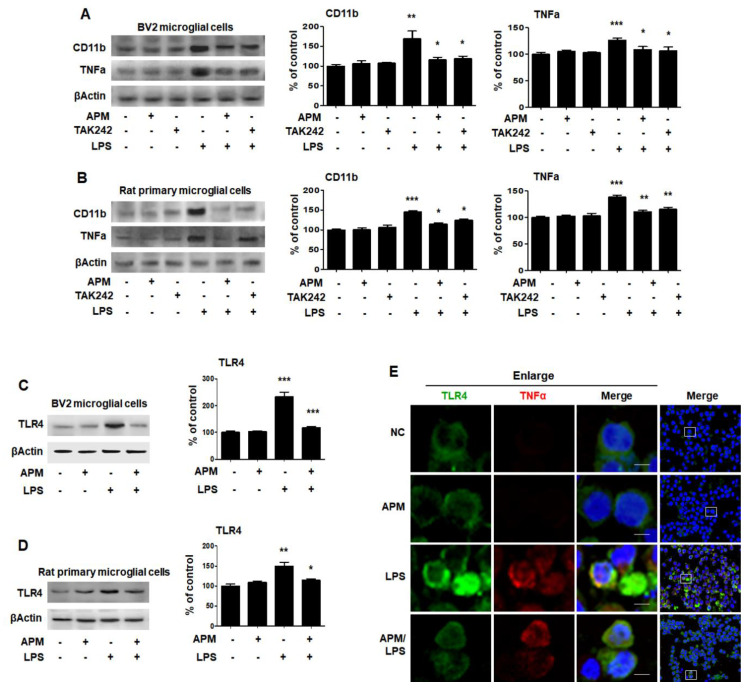
APM inhibits LPS-induced CD11b and TNFα expression by inhibiting TLR4 in BV and rat primary microglial cells. Cells were treated with APM for 1 h followed by LPS for 12 h. CD11b and TNFα expression were significantly inhibited in LPS-stimulated BV2 (**A**) and rat primary microglial cells (**B**) by TLR4 inhibitor, TAK242. Cells were treated with APM for 1 h followed by LPS for 6 h. APM significantly reduced LPS-induced TLR4 expression in BV2 (**C**) and rat primary microglial cells (**D**). Immunofluorescence double staining for TLR4 (green) and TNFα (red) localization (**E**) in BV2 microglial cells. Cell were counterstained with DAPI (blue). Magnification 400×. Enlarge figure of scale bars: 5 μm. βActin was used to confirm equal sample loading. CD11b, TNFα and TLR4 followed by densitometric analysis. The data are representative of three independent experiments and quantified as mean values ± SEM. Tukey’s multiple comparison test, * *p* < 0.05, ** *p* < 0.01, *** *p* < 0.001 compared to normal control.

LPS binds to TLR4 on the surface of microglial cells to increase immune responses [30]. Therefore, we investigated whether APM can modulate the proinflammatory response through LPS and TLR4 interactions at the cell surface. BV2 and rat primary microglial cells were treated with TAK242 for 1 h followed by LPS for 12 h, and then immunoblotting and immunofluorescence staining were performed. TAK242 and APM significantly decreased LPS-induced CD11b and TNFα expression in BV2 and rat primary microglial cells (Figure 3A,B). In addition, APM significantly reduced LPS-induced TLR4 expression in BV2 and rat primary microglial cells (Figure 3C,D). To further confirm our findings, we observed the subcellular localization of TNFα and TLR4. APM clearly inhibited TLR4 and TNFα subcellular localization in LPS-stimulated BV2 microglial cells (Figure 3E). These results suggest that APM can alter the LPS-induced proinflammatory response in microglial cells by inhibiting the interaction between LPS and TLR4.

### 2.4. APM Suppresses LPS-Induced Proinflammatory Cytokines by Inhibiting Translocation of p65/STAT3 and MAPK-ERK Signaling in Microglial Cells

**Figure 4:**
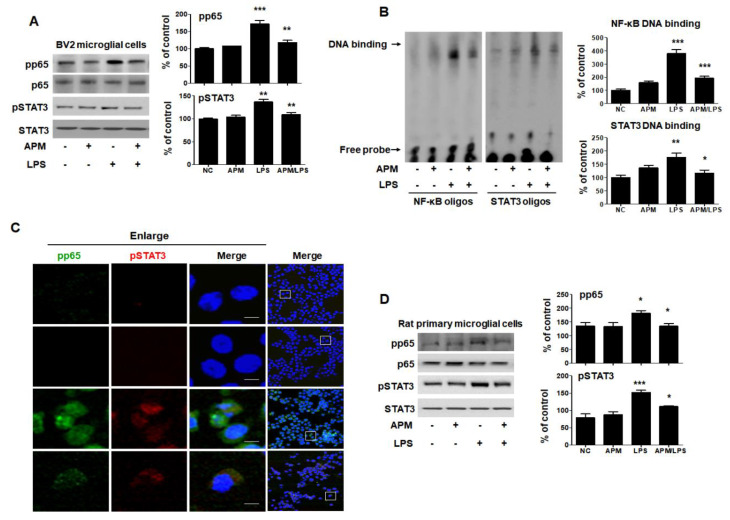
APM suppresses translocation of p65 and STAT3 in LPS-stimulated BV2 and rat primary microglial cells. Cells were treated with APM for 1 h followed by LPS for 6 h. Expression of pp65 and pSTAT3 in nuclear extracts was measured by immunoblotting in BV2 and rat primary microglial cells. APM significantly reduced LPS-induced phosphorylation of p65 and pSTAT3 in BV2 (**A**) and rat primary microglial cells (**D**). DNA-binding activity of NF-κB and STAT3 in nuclear extracts was measured by electrophoretic mobility shift analysis (EMSA) in BV2 microglial cells (**B**). EMSA was quantified by densitometric analysis. Immunofluorescence double staining for pp65 (green) and pSTAT3 (red) localization (**C**) in BV2 microglial cells. Cell were counterstained with DAPI (blue). Magnification 400×. Enlarge figure of scale bars: 5 μm. βActin was used to confirm equal sample loading. pp65 and pSTAT3 followed by densitometric analysis. The data are representative of three independent experiments and quantified as mean values ± SEM. Tukey’s multiple comparison test, * *p* < 0.05, ** *p* < 0.01, *** *p* < 0.001 compared to normal control.

The transcription factor NF-κB p65 and activated STAT3 are key signaling molecules that regulate the proinflammatory cytokine level following LPS treatment [31,32]. Therefore, we examined the translocation of NF-κB p65 and STAT3 in response to LPS-induced neuroinflammation to BV2 microglial cells. BV2 and rat primary microglial cells were pretreated with APM for 1 h and then stimulated with LPS for 6 h. Along with phosphorylation, NF-κB-p65 and STAT3 were translocated from the cytoplasm to the nucleus after LPS stimulation; however, this was effectively inhibited by APM (Figure 4A). We further evaluated the effects of APM on NF-κB and STAT3′s DNA-binding activity when treated with LPS (Figure 4B). NF-κB-DNA and STAT3-DNA complex in LPS-stimulated BV2 microglial cells were strongly up-regulated; however, the increase in these complexes was significantly suppressed by APM treatment. These results are further supported by our observations of the intracellular localization of NF-κB and STAT3 distributed in the nuclear compartment of untreated cells that act as transcriptional activators. When cells were treated with APM, phosphorylation of NF-κB and STAT3 proteins seemed to be predominantly localized in the cytoplasm rather than accumulating in the nucleus (Figure 4C). In addition, a decrease in TNF expression was observed according to NF-κB and STAT3 translocations. Lastly, we tested whether APM alters LPS-stimulated rat primary microglial cells. Rat primary microglial cells were treated with APM for 1 h followed by LPS for 6 h. We found that APM treatment significantly decreased LPS-induced phosphorylation and translocation of NF-κB and STAT3 in rat primary microglial cells (Figure 4D). The results were entirely consistent with our earlier data.

**Figure 5:**
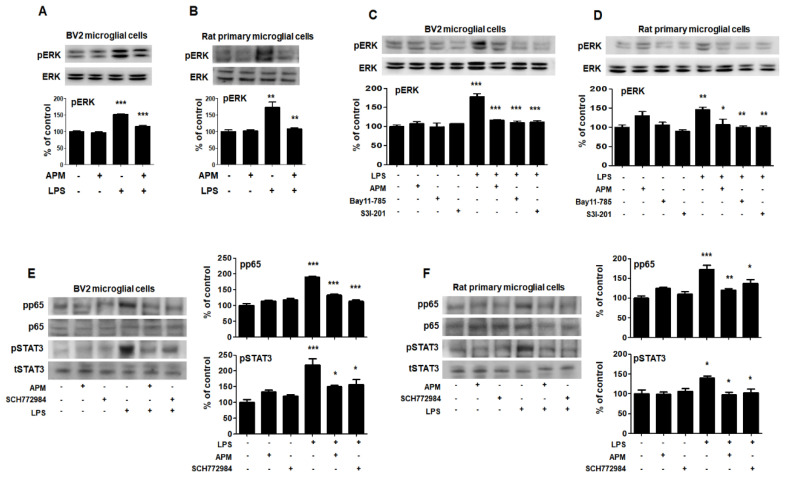
APM inhibits LPS-induced MAPK-ERK phosphorylation and interaction of MAPK-ERK and p65/STAT3 in BV2 and rat primary microglial cells. Cells were treated with APM for 1 h followed by LPS for 6 h. LPS-induced MAPK-ERK phosphorylation was inhibited by APM administration in BV2 (**A**) and rat primary microglial cells (**B**). Inhibitors of NF-κB (10 μM Bay11-7085), STAT3 (10 μM S3I-201) or APM strongly suppressed LPS-induced MAPK-ERK phosphorylation in BV2 (**C**) and rat primary microglial cells (**D**). MAPK-ERK inhibitor (10 μM SCH772984) or APM effectively suppressed LPS-induced p65 and STAT3 phosphorylation in BV2 (**E**) and rat primary microglial cells (**F**). βActin was used to confirm equal sample loading. pERK, pp65, and pSTAT3 followed by densitometric analysis. The data are representative of three independent experiments and quantified as mean values ± SEM. Tukey’s multiple comparison test, * *p* < 0.05, ** *p* < 0.01, *** *p* < 0.001 compared to normal control.

The roles of MAPKs in the production of LPS-induced proinflammatory mediators have been well documented [14]. Therefore, we aimed to evaluate whether MAPK pathways were associated with the SK channel and neuroinflammatory activities of APM in BV2 microglial cells. As shown in Figure 5A and Figure S2, phosphorylated-ERK/c-Jun NH2-terminal kinase (JNK)/p38 was significantly induced in LPS-stimulated BV2 microglial cells. APM inhibited LPS-induced phosphorylation of ERK and JNK. Interestingly, APM strongly inhibited LPS-induced phosphorylation of ERK. The results of this interesting inhibition of ERK phosphorylation were also confirmed in LPS-stimulated rat primary microglial cells (Figure 5B). The control effect of APM’s specific ERK is consistent with our other reports [22].

To confirm our finding that APM inhibits LPS-induced SK channels and proinflammatory activities through p65, STAT3, and MAPK-ERK signaling, we performed the following experiment after treating cells with inhibitors of p65 (Bay11-7085), STAT3 (S3I-201), and ERK (SCH772984). These inhibitors significantly decreased LPS-induced CD11b, TNFα, TLR4, and pCaMKⅡ expression in BV2 and rat primary microglial cells (Figure S3). 

To further investigate the interaction role of in LPS-induced phosphorylation of p65/STAT3 and MAPK-ERK, cells were treated with Bay11-7085, S3I-201, SCH772984, and APM. LPS-induced phosphorylation of MAPK-ERK was significantly inhibited by Bay11-7085, S3I-201, and APM in BV2 microglial cells (Figure 5C). These results were alco consistent with those of LPS-stimulated rat primary microglial cells (Figure 5D). In addition, LPS-induced phosphorylation of p65/STAT3 was significantly inhibited by SCH772984 and APM in BV2 microglial cells (Figure 5E). These results were alco consistent with those of LPS-stimulated rat primary microglial cells (Figure 5F). Together, these data confirm the interaction between MAPK-ERK phosphorylation and p65/STAT3 translocation in activated microglia, suggesting that APM plays an important role as their modulator.

## 3. Discussion

Microglia have been known to play an important role in neurodegenerative diseases [33]. Increasing evidence suggests that controlling the activation of microglia may have protective effects against neurodegenerative diseases [5].

The microglial proinflammatory response is attributed to the activation of Ca^2+^-activated SK channels and TLR4 by LPS, which triggers downstream inflammatory signaling pathways, leading to protein phosphorylation, nuclear translocation, and, ultimately, to proinflammatory cytokines mediators [4,6,8,18,30,34]. During pathological conditions associated with CNS inflammation, proinflammatory cytokines are representative targets of activated microglia [5] and targeting them is suggested to have therapeutic benefits.

APM, one of the components of bee venom, has been suggested to possess beneficial effects in the treatment of PD [35,36,37]. We have reported the anti-inflammatory, antifibrotic, and anticancer properties of bee venom and its major components, melittin and apamin [22,23,24,25,26,38,39,40]. Studies have investigated the physiological function of APM; however, the molecular mechanisms of the LPS-induced proinflammatory and cellular signaling potential of APM in neuroinflammation have not yet been elucidated. The major findings of this study are that APM suppresses LPS-induced SK channels and TLR4 expression through the inhibition of NF-κB and STAT3 translocation and ERK phosphorylation and that it finally suppresses neuroinflammatory responses in microglia.

Proinflammatory cytokines such as TNFα, IL1β, IL-6, and COX2 play an important role in the pathological process of neurodegenerative diseases [41,42]. Several studies have suggested that the up-regulation of TNFα, IL1β, IL6, and COX2 plays important roles in many inflammatory diseases including cytotoxicity in brain injuries and many neurodegenerative diseases such as AD, PD, and prion diseases [5,43,44]. Thus, many studies have been conducted to find their inhibitors [9,14,15,17,28,31,32]. Our results showed that APM reduced the expression of TNFα, IL1β, IL6, and COX2 by blocking their expression in LPS-stimulated BV2 and rat primary microglial cells. These findings are similar to the results of the release and mRNA level of TNFα, IL1β, and IL6. The results indicated that APM has an anti-inflammatory effect on microglia; this is consistent with our other research reports [24,25].

LPS is considered a powerful stimulant of the proinflammatory response in microglia, and its polynomial action can be triggered through SK channels and TLR4 [2,8]. SK channels are associated with neuroinflammation in microglia. The CaMKII pathway was found to be significantly activated in LPS-stimulated microphages, with an increase in intracellular Ca^2+^ levels and CaMKII phosphorylation [29,45]. APM, a specifically selective blocker of SK channels, decreased LPS-induced SK/K_Ca_2.2 and pCaMKII expression in BV2 and rat primary microglial cells.

Microglia can recognize pathogen-associated molecular patterns through TLR pattern recognition receptors expressed on the cell surface [46]. TLR4 is known to recruit adapter molecules, and LPS mediates immune activation [28]. CaMKII promotes the production of TLR-triggered proinflammatory cytokines in macrophages [47]. In the present study, TAK242 and APM decreased LPS-induced CD11b and TNFα expression in BV2 and rat primary microglial cells. In addition, APM alters the LPS-induced TLR4 expression in BV2 and rat primary microglial cells. Overall, APM alters the activity of microglia by blocking the SK channel and TLR4 through the polynomial action by LPS. These results are related to a decrease in neuroinflammatory responses through the SK channel and TLR4 reduced by APM in microglia.

The interaction between LPS and SK channel/TLR4 activates the NF-κB, STAT, and MAPK signaling pathways in microglia [8,15,17,31]. Activated NF-κB and STAT were reported to play roles in the regulation of neuroinflammatory responses [11,17]. The potential involvement of NF-κB and STAT3 was investigated because the promoters of both TNFα and IL-6 contain binding sites for NF-κB and STAT3, which are known to be involved in neurological disorders associated with increased inflammation [11,32,48]. Recently, several studies have reported that the cell signaling control of these transcription factors inhibits neuroinflammation [13,14,15,17,28]. Our previous studies reported anti-inflammatory effects in dopaminergic neurons, mesencephalic neurons, and BV2 microglial cells through the strong inhibition of NF-κB and STAT3 translocation [19]. In addition, our other study reported that APM inhibits inflammatory cytokines and chemokines through the suppression of NF-κB and STAT [24]. In accordance with these findings, our results showed that APM effectively inhibits NF-κB and STAT3 in LPS-stimulated BV2 and rat primary microglial cells.

MAPK-ERK signaling is associated with LPS-induced immune responses via SK channels and TLR4 signaling [3,28,30]. In addition, this signaling maintains NF-κB and STAT3 signaling in microglia [17,19]. In this study, APM also strongly suppressed LPS-induced ERK phosphorylation in BV2 and rat primary microglial cells. Thus, APM is believed to be a strong inhibitor of neuroinflammation by regulating mediated to increased SK channels and TLR4-mediated signaling pathways in microglia.

Collectively, these results suggest that APM is a potent inhibitor of neuroinflammatory mechanisms and may therefore potentially find therapeutic applications for a wide range of pathological conditions, including neurological diseases.

## 4. Materials and Methods

### 4.1. Cell Cultures and Conditions

BV-2 murine microglia cells, a generous gift from Dr Hoe (Korea Brain Research Institute, KBRI), were maintained in Dulbecco’s Modified Eagle’s Medium (DMEM) medium Gibco, (Grand Island, NY, USA) containing 5% fetal bovine serum (Gibco) and 0.1% gentamicin (Gibco) in a 5% CO_2_ incubator. 

Rat primary microglial cells were prepared from the cerebral cortices of 1-day-old Sprague Dawley rat, as described previously [49]. All experimental procedures used in the current study were approved by the institutional animal care and use committee at the Daegu Catholic University Medical Center (EXP-IRB number: DCIAFCR-191112-07-Y). To separate the cortices into single cells, use a high-glucose DMEM containing 10% FBS/penicillin-streptomycin solution (Gibco) and plated into 75 T culture flasks. Then, the rat primary microglial cells were harvested by shaking the plate for 2 h at 120 rpm. Rat primary microglia cells were resuspended at 1 × 10^5^ cells per well to poly-D-lysine (Sigma-Aldrich, St Louis, MO, USA) coated 12 well plates. 

### 4.2. Cytotoxicity Assay

To evaluate the effect of APM on LPS-stimulated proliferation of microglia, BV2 microglial cells were plated in 96-well culture plates at 1 × 10^5^ cells/mL in culture medium and allowed to attach for 12 h. Media were then discarded and replaced with new medium containing various concentrations of APM. Cell viability was analyzed using the Cell Counting Kit (CCK-8; Dojindo Laboratories, Kumamoto, Japan) assay according to the manufacturer’s instructions. The absorbance at 450 nm was assessed using a microplate reader (Thermo Fisher Scientific, Waltham, MA, USA).

### 4.3. Treatment Kinase Inhibitors 

Cells were pretreated with various inhibitors such as TLR4-specific inhibitor (Millipore, Bedford, MA, USA): TAK242 (500 nM), NF-κB-specific inhibitor (Sigma-Aldrich): Bay11-7085 (10 μM), STAT3-specific inhibitor (Sigma-Aldrich): S3I-201 (10 μM) and ERK1/2-specific inhibitor (Cell signaling, Danvers, MA, USA): PD98059 (10 μM). After 1 h, the cells were treated and co-cultured with LPS for 6 h or 12 h.

### 4.4. Enzyme-Linked Immunosorbent Assay (ELISA)

The culture medium of the cells was harvested, and cytokine production (TNFα, IL1β and IL6) in the supernatant was measured with a solid phase sandwich ELISA using a Quantikine mouse TNFα, mouse IL1β and mouse IL6 kit (R&D systems, Minneapolis, MN, USA) according to the manufacturer’s instructions.

### 4.5. Quantitative Real-Time Polymerase Chain Reaction (PCR) Analysis

mRNA transcription of cytokines was analyzed by qRT-PCR. Total RNA was extracted from BV2 microglial cells using TRIzol Reagent (Thermo Fisher Scientific) according to the manufacturer’s recommendations. Reverse transcription reaction was performed using EcoDry Premix Kit (TaKaRa, Tokyo, Japan). cDNA was subjected to qRT-PCR using SYBR Green Mix kit (Toyobo, Osaka, Japan) and the CFX Connect real-time PCR system (Bio-Rad Laboratories, Hercules, CA, USA). Primers, synthesized at Microgen (Daejon, Korea), were as follows: TNF-α: forward (F), 5′-GAT TAT GGC TCA GGG TCC AA-3′, reverse (R), 5′-GCT CCA GTG AAT TCG GAA AG-3′; IL1β: F’, 5′-AGC TGG AGA GTG TGG ATC CC-3′, R, 5′-CCT GTC TTG GCC GAG GAC TA-3′; IL-6: F′, 5′-CCA CTT CAC AAG TCG GAG GC-3′, and R′, 5′-GGA GAG CAT TGG AAA TTG GGG T-3′; GAPDH: F′, CAG GAG CGA GAC CCC ACT AA, and R′, ATC ACG CCA CAG CTT TCC AG. GAPDH is served as a normalization control. The relative RNA expression of each gene was analyzed using the 2^−ΔΔ*C*T^ method as previously reported [50].

### 4.6. Immunoblot Analysis

Protein samples were prepared from the cultured BV2, primary microglial and primary astrocyte cells with a protein extraction buffer (Cell Lytic™ M; Sigma) and NE-PER Nuclear and Cytoplasmic Extraction Kit (Thermo Fisher Scientific) according to the instruction manual. The protein extract concentration of cells was measured with Bradford assay (Bio-Rad Laboratories) by using a spectrophotometer. The protein samples were loaded on precast gradient polyacrylamide gels (Bolt™ 4–12% Bis-Tris Plus Gels; Thermo Fisher Scientific) and transferred to nitrocellulose membranes (GE Healthcare, Madison, WI, USA) by using Bolt™ Mini Blot Module and Mini Gel Tank (Thermo Fisher Scientific), according to the manufacturer’s recommendations. The membrane blocked with 5% bovine serum albumin was probed with a primary antibody and horseradish peroxidase-conjugated secondary antibody. The chemiluminescent substrate (Thermo Fisher Scientific) was used to detect the protein bands. Image analyses were performed using the ChemiDoc™ XRS+ (Bio-Rad Laboratories). 

The resolved proteins were transferred onto a nitrocellulose membrane and probed with anti-TNFα (Abcam, Cambridge, MA, USA), anti-IL1β (Abcam), anti-IL6 (Abcam), anti-COX2 (Santa Cruz Biotechnology, Dallas, TX, USA), anti-CD11b (Cell signaling), anti-K_Ca_2.2 (Alomone labs, Jerusalem, Israel), anti-pCaMKII (Novus, Littleton, CO, USA), anti-CaMKII (Novus), anti-TLR4 (Santa Cruz Biotechnology), anti-pp65 (Cell signaling), anti-p65 (Cell signaling), anti-pSTAT3 (Cell signaling), anti-STAT3 (Cell signaling), anti-pERK (Cell signaling), anti-ERK (Cell signaling), anti-pJNK (Cell signaling), anti-JNK (Cell signaling), anti-pp38 (Cell signaling), anti-p38 (Cell signaling), and anti-βActin (Sigma-Aldrich).

### 4.7. Cytospin Preparation and Immunofluorescent Staining

Microglial cells were placed on coated microscope slides using cytocentrifugation 5 min at 500 rpm) and fixed with 3.7% paraformaldehyde in PBS. The fixed cells were incubated with primary antibodies for 1 h. After washing, they were incubated with the secondary antibodies (Alexa Flour 488 or 594, Invitrogen, Carlsbad, CA, USA). Nuclear staining was performed with DAPI (ImmunoChemistry, Bloomington, MN, USA). The mounting of slide was using ProLong^®^ Gold antifade reagent (Molecular Probes^®^ by Life Technologies^TM^, Carlsbad, CA, USA).

Primary antibodies used are following anti-CD11 (Cell signaling), anti-TNFα (Abcam), anti-pCaMKII (Novus), anti-TLR4 (Santa Cruz Biotechnology), anti-pp65 (Cell signaling), and anti-pSTAT3 (Cell signaling). Immunolabeling was examined using an Eclipse Ti-U and confocal microscope (Nikon, Tokyo, Japan).

### 4.8. Electrophoretic Mobility Shift Analysis

Nuclear extract fractionation from BV2 primary microglial cells were conducted using an NE-PER Nuclear and Cytoplasmic Extraction Kit (Thermo Fisher Scientific) according to the manufacturer’s instructions. The lightshift chemiluminescent electrophoretic mobility shift analysis (EMSA) Kit (Thermo Fisher Scientific) was used for the EMSA to analyze the expression of NF-κB and STAT3. The consensus NF-κB binding site (5′-AGT TGA GGG GAC TTT CCC AGG C-3′) and STAT3 binding site (5′-CTT CAT TTC CCG TAA ATC CCT AAA GCT-3′) were used as DNA-binding oligos.

### 4.9. Statistical Analysis

All data analysis was performed with the GraphPad Prism 5 (GraphPad Software, Inc., San Diego, CA) using either a one-way ANOVA with Tukey’s post hoc test for multiple comparisons and data are presented as the mean ± S.E.M. (* *p* < 0.05, ** *p* < 0.01, *** *p* < 0.001). 

## Supplementary Materials

The following are available online at https://www.mdpi.com/1422-0067/21/12/4319/s1. Figure S1: Increased inflammatory cytokines by LPS and cell viability of APM. Figure S2: Effect of APM on LPS-induced MAPK-JNK and p38 phosphorylation. Figure S3: Inhibitors of p65 (Bay11-7085), STAT3 (S3I-201), and ERK (SCH772984) inhibits LPS-induced CD11b, TNFα, TLR4, and pCaMKII expression in BV2 and rat primary microglial cells.

## Abbreviations

APM	Apamin
SK	Small conductance calcium-activated potassium
LPS	Lipopolysaccharide
TLR4	Toll-like receptor
NF-κB	Nuclear factor kappa-light-chain-enhancer of activated B cells
STAT	Signal transducer and activator of transcription
MAPK	Mitogen-activated protein kinases
ERK	Extracellular signal-regulated kinase
AD	Alzheimer’s disease
PD	Parkinson’s disease
CNS	Central nervous system
TNF	Tumor necrosis factor
IL	Interleukin
COX	Cyclooxygenases
IFN	Interferon
