# Supplementary material for: Apamin Suppresses LPS-Induced Neuroinflammatory Responses by Regulating SK Channels and TLR4-Mediated Signaling Pathways
Source: Int J Mol Sci. 2020 Jun 17;21(12):4319. doi: 10.3390/ijms21124319 (PMC7352249; doi:10.3390/ijms21124319)

Figure S1: Increased inflammatory cytokines by LPS and cell viability of APM. BV2 microglial cells were treated with various concentration of LPS and APM for 12 h. Secretion of TNFα (A), expression of TNFα (B) and microglial cells marker, CD11b were significantly induced in LPS-stimulated BV2 microglial cells. (C) Dose-dependent effect of APM on the viability of BV2 microglial cells. βActin was used to confirm equal sample loading. TNFα and CD11b followed by densitometric analysis. The data are representative of three independent experiments and quantified as mean values ± SEM. Tukey's multiple comparison test, * $p < 0.05$, ** $p < 0.01$, *** $p < 0.001$ compared to normal control.

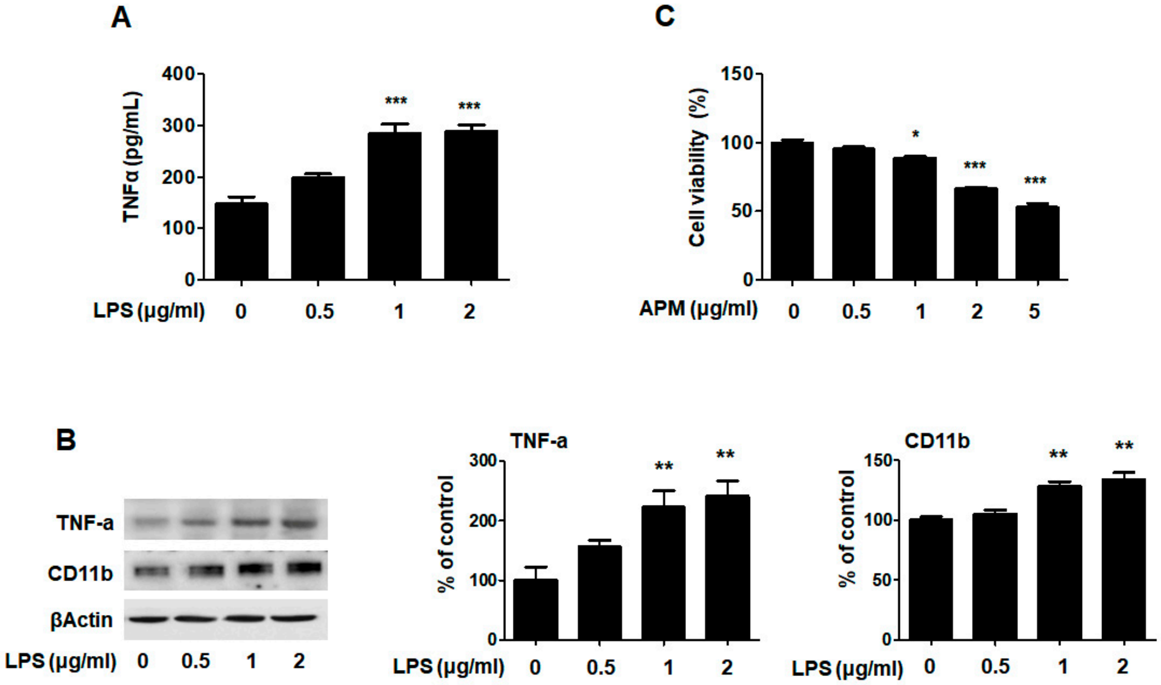

Figure S2: Effect of APM on LPS-induced MAPK-JNK and p38 phosphorylation. The data are representative of three independent experiments and quantified as mean values ± SEM. Tukey's multiple comparison test, * $p < 0.05$, ** $p < 0.01$, *** $p < 0.001$ compared to normal control.

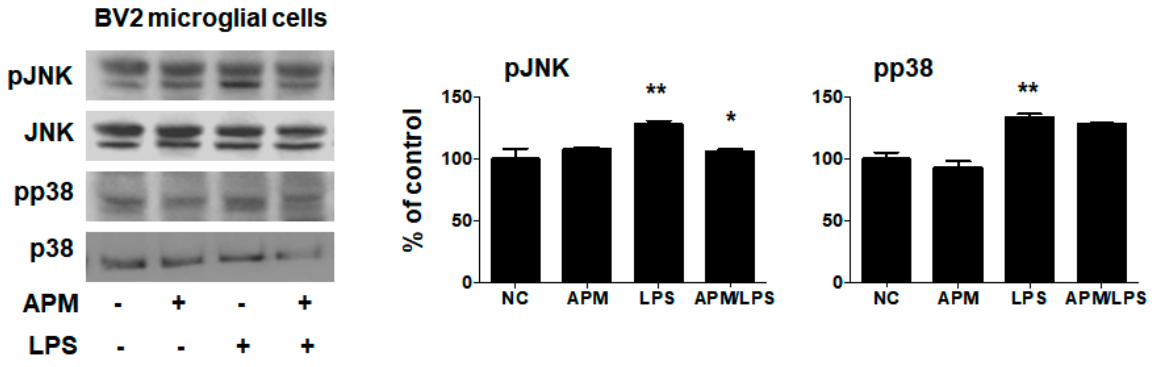

Figure S3: Inhibitors of p65 (Bay11-7085), STAT3 (S3I-201), and ERK (SCH772984) inhibits LPS-induced CD11b, TNFα, TLR4, and pCaMKII expression in BV2 and rat primary microglial cells. βActin was used to confirm equal sample loading. The data are representative of three independent experiments and quantified as mean values ± SEM. Tukey's multiple comparison test, * $p < 0.05$, ** $p < 0.01$, *** $p < 0.001$ compared to normal control.

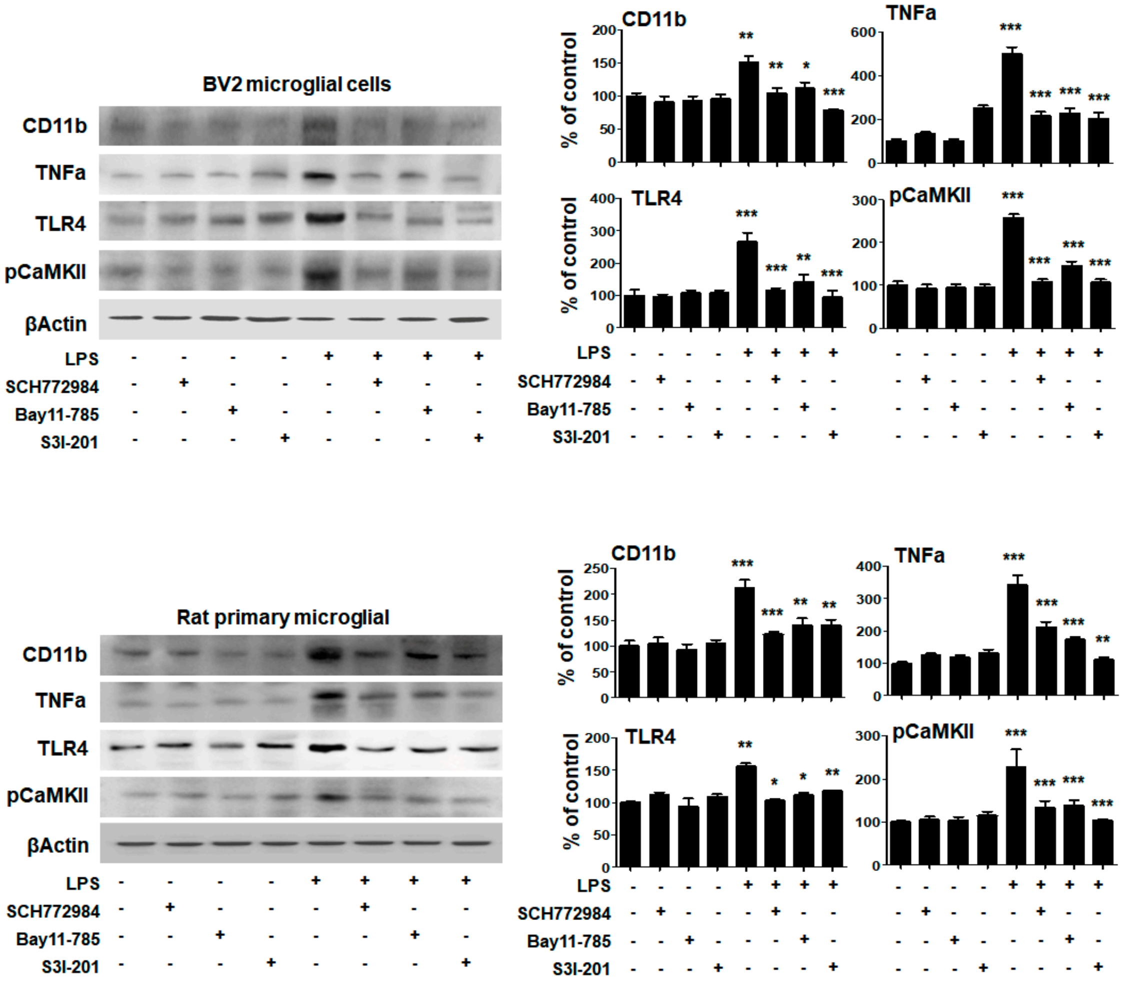

Supplement: Supplementary file 1 [file ijms-21-04319-s001.pdf]
